# Supplementary material for: SARS-CoV-2 variant survey: Comparison of RT-PCR screening with TGS and variant distribution across two divisions of Bangladesh
Source: PLoS One. 2024 Oct 17;19(10):e0311993. doi: 10.1371/journal.pone.0311993 (PMC11486398; doi:10.1371/journal.pone.0311993)
Supplement: S1 Table — (DOCX) [file pone.0311993.s001.docx]

**S 1 Table:**

**Table: Specimen ID and corresponding GenBank accession ID for 99 sequences**

| # | Sample ID | GenBank Accession | Date of sample collection (YYYY-MM-DD) |
| --- | --- | --- | --- |
| 1 | BD_IVPP_171 | OR889496 | 2021-04-14 |
| 2 | BD_IVPP_174 | OR889497 | 2021-04-17 |
| 3 | BD_B_210531_119 | OR889498 | 2021-05-31 |
| 4 | BD_B_200522_22 | OR889499 | 2020-05-22 |
| 5 | BD_B_210521_38 | OR889500 | 2021-05-21 |
| 6 | BD_B_210528_57 | OR889501 | 2021-05-28 |
| 7 | BD_B_210630_35 | OR889502 | 2021-06-30 |
| 8 | BD_B_210730_122 | OR889503 | 2021-07-30 |
| 9 | BD_B_210702_55 | OR889504 | 2021-07-02 |
| 10 | BD_B_210707_653 | OR889505 | 2021-07-07 |
| 11 | BD_B_210823_228 | OR889506 | 2021-08-23 |
| 12 | BD_B_210828_438 | OR889507 | 2021-08-28 |
| 13 | BD_B_210904_394 | OR889508 | 2021-09-04 |
| 14 | BD_B_211115_407 | OR889509 | 2021-11-15 |
| 15 | BD_B220113_03 | OR889510 | 2022-01-13 |
| 16 | BD_B220123_60 | OR889511 | 2022-01-23 |
| 17 | BD_B_220125_93 | OR889512 | 2022-01-25 |
| 18 | BD_B_220129_126 | OR889513 | 2022-01-29 |
| 19 | BD_B_220203_164 | OR889514 | 2022-02-03 |
| 20 | BD_IVPP_076 | OR889515 | 2021-11-27 |
| 21 | BD_IVPP_072 | OR889516 | 2021-10-19 |
| 22 | BD_IVPP_180 | OR889517 | 2021-06-17 |
| 23 | BD_IVPP_188 | OR889518 | 2021-06-07 |
| 24 | BD_IVPP_275 | OR889519 | 2021-08-14 |
| 25 | BD_IVPP_274 | OR889520 | 2021-08-11 |
| 26 | BD_IVPP_212 | OR889521 | 2021-04-01 |
| 27 | BD_IVPP_217 | OR889522 | 2021-04-06 |
| 28 | BD_IVPP_221 | OR889523 | 2021-03-30 |
| 29 | BD_IVPP_063 | OR889524 | 2021-08-12 |
| 30 | BD_IVPP_068 | OR889525 | 2021-09-13 |
| 31 | BD_IVPP_035 | OR889526 | 2021-12-26 |
| 32 | BD_IVPP_234 | OR889527 | 2020-11-12 |
| 33 | BD_IVPP_074 | OR889528 | 2021-11-19 |
| 34 | BD_IVPP_177 | OR889529 | 2021-04-24 |
| 35 | BD_IVPP_208 | OR889530 | 2021-05-15 |
| 36 | BD_B_220205_325 | OR889531 | 2022-02-05 |
| 37 | BD_B_220212_120 | OR889532 | 2022-02-12 |
| 38 | BD_B_220214_356 | OR889533 | 2022-02-14 |
| 39 | BD_B_220208_95 | OR889534 | 2022-02-08 |
| 40 | BD_B_220205_390 | OR889535 | 2022-02-05 |
| 41 | BD_IVPP_040 | OR889536 | 2021-08-01 |
| 42 | BD_B_200426_211 | OR889537 | 2020-04-26 |
| 43 | BD_B_220609_14 | OR889538 | 2022-06-09 |
| 44 | BD_IVPP_210 | OR889539 | 2021-05-27 |
| 45 | BD_IVPP_230 | OR889540 | 2020-12-31 |
| 46 | BD_IVPP_218 | OR889541 | 2021-07-04 |
| 47 | BD_B_220305_22 | OR889542 | 2022-03-05 |
| 48 | BD_IVPP_289 | OR889543 | 2022-06-20 |
| 49 | BD_IVPP_020 | OR889544 | 2021-12-23 |
| 50 | BD_IVPP_037 | OR889545 | 2021-12-29 |
| 51 | BD_IVPP_264 | OR889546 | 2022-02-07 |
| 52 | BD_IVPP_267 | OR889547 | 2022-01-20 |
| 53 | BD_IVPP_285 | OR889548 | 2022-06-13 |
| 54 | BD_IVPP_288 | OR889549 | 2022-06-15 |
| 55 | BD_IVPP_233 | OR889550 | 2020-11-02 |
| 56 | BD_IVPP_238 | OR889551 | 2022-12-08 |
| 57 | BD_IVPP_240 | OR889552 | 2020-12-26 |
| 58 | BD_IVPP_211 | OR889553 | 2021-05-31 |
| 59 | BD_IVPP_145 | OR889554 | 2021-08-12 |
| 60 | BD_IVPP_147 | OR889555 | 2021-12-08 |
| 61 | BD_IVPP_290 | OR889556 | 2022-06-20 |
| 62 | BD_IVPP_291 | OR889557 | 2022-06-23 |
| 63 | BD_IVPP_292 | OR889558 | 2022-06-24 |
| 64 | BD_IVPP_293 | OR889559 | 2022-06-25 |
| 65 | BD_IVPP_294 | OR889560 | 2022-06-26 |
| 66 | BD_IVPP_296 | OR889561 | 2022-06-29 |
| 67 | BD_IVPP_297 | OR889562 | 2022-06-30 |
| 68 | BD_IVPP_298 | OR889563 | 2022-07-02 |
| 69 | BD_IVPP_299 | OR889564 | 2022-07-03 |
| 70 | BD_IVPP_300 | OR889565 | 2022-07-04 |
| 71 | BD_B_220118_239 | OR889566 | 2022-01-18 |
| 72 | BD_B_210626_07 | OR889567 | 2021-06-26 |
| 73 | BD_B_220612_03* |  | 2022-06-12 |
| 74 | BD_B_220613_41 | OR889568 | 2022-06-13 |
| 75 | BD_B_220118_269 | OR889569 | 2022-01-18 |
| 76 | BD_B_220109_357 | OR889570 | 2022-01-09 |
| 77 | BD_B_220107_01 | OR889571 | 2022-01-07 |
| 78 | BD_B_211212_05 | OR889572 | 2021-12-12 |
| 79 | BD_B_211228_12 | OR889573 | 2021-12-28 |
| 80 | BD_B_210627_203 | OR889574 | 2021-06-27 |
| 81 | BD_B_220613_36 | OR889575 | 2022-06-13 |
| 82 | BD_B_220507_01 | OR889576 | 2022-05-07 |
| 83 | BD_B_210904_47 | OR889577 | 2021-09-04 |
| 84 | BD_B_220111_03 | OR889578 | 2022-01-11 |
| 85 | BD_B_220610_04 | OR889579 | 2022-06-10 |
| 86 | BD_B_220309_04 | OR889580 | 2022-03-09 |
| 87 | BD_B_220215_349 | OR889581 | 2022-02-15 |
| 88 | BD_B_210823_05 | OR889582 | 2021-08-23 |
| 89 | BD_IVPP_179 | OR889583 | 2021-06-06 |
| 90 | BD_B_210311_183 | OR889584 | 2021-03-11 |
| 91 | BD_B_210313_10 | OR889585 | 2021-03-13 |
| 92 | BD_B_210717_94 | OR889586 | 2021-07-17 |
| 93 | BD_B_210310_03 | OR889587 | 2021-03-10 |
| 94 | BD_IVPP_167 | OR889588 | 2021-04-12 |
| 95 | BD_IVPP_286 | OR889589 | 2022-06-13 |
| 96 | BD_IVPP_287 | OR889590 | 2022-06-14 |
| 97 | BD_IVPP_295 | OR889591 | 2022-06-29 |
| 98 | BD_B_220103_03 | OR889592 | 2022-01-03 |
| 99 | BD_B_211014_06 | OR889593 | 2021-10-14 |

*Accession ID could not be recovered for this specimen.
