# Supplementary material for: SARS-CoV-2 variant survey: Comparison of RT-PCR screening with TGS and variant distribution across two divisions of Bangladesh
Source: PLoS One. 2024 Oct 17;19(10):e0311993. doi: 10.1371/journal.pone.0311993 (PMC11486398; doi:10.1371/journal.pone.0311993)
Supplement: S2 Table — (DOCX) [file pone.0311993.s002.docx]

**S5 Table:**

**Table:** **Probable SARS-CoV-2 variants detected by variant RT-PCR from April 2020 to July 2022 in Dhaka and Chattogram, color-coded as frequency distributions**

| **Time frame** | **Wild-type** | **Alpha** | **Beta** | **Delta** | **Kappa** | **Omicron** | **Zeta** | **Undetermined** | **Total** | **Corresponding predominant global variant*** |
| --- | --- | --- | --- | --- | --- | --- | --- | --- | --- | --- |
| Apr 20 | 6 | 0 | 0 | 0 | 0 | 0 | 0 | 0 | 6 | Wild type |
| May 20 | 7 | 0 | 0 | 0 | 0 | 0 | 0 | 0 | 7 | Wild type |
| June 20 | 5 | 0 | 0 | 0 | 0 | 0 | 0 | 0 | 5 | Wild type |
| Nov 20 | 3 | 0 | 0 | 0 | 0 | 0 | 0 | 1 | 4 | Alpha and Beta |
| Dec 20 | 5 | 0 | 0 | 0 | 0 | 0 | 0 | 2 | 7 | Alpha and Beta |
| Jan 21 | 0 | 0 | 0 | 0 | 0 | 0 | 0 | 5 | 5 | Alpha and Beta |
| Mar 21 | 0 | 26 | 9 | 0 | 0 | 0 | 0 | 1 | 36 | Alpha and Beta |
| Apr 21 | 0 | 2 | 15 | 0 | 0 | 0 | 1 | 0 | 18 | Gamma |
| May 21 | 0 | 1 | 11 | 3 | 1 | 0 | 1 | 0 | 17 | Gamma |
| Jun 21 | 0 | 1 | 0 | 38 | 0 | 0 | 0 | 0 | 39 | Delta |
| Jul 21 | 0 | 0 | 0 | 47 | 0 | 0 | 0 | 0 | 47 | Delta |
| Aug 21 | 0 | 0 | 0 | **53** | 0 | 0 | 0 | 0 | **53** | Delta |
| Sep 21 | 0 | 0 | 0 | 25 | 0 | 0 | 0 | 0 | 25 | Delta |
| Oct 21 | 0 | 0 | 0 | 18 | 0 | 0 | 0 | 0 | 18 | Delta |
| Nov 21 | 0 | 0 | 0 | 17 | 0 | 0 | 0 | 0 | 17 | Delta |
| Dec 21 | 0 | 0 | 0 | **69** | 7 | 4 | 0 | 0 | **80** | Omicron (B.1.1.529) |
| Jan 22 | 0 | 0 | 0 | 9 | 0 | **109** | 0 | 0 | **118** | Omicron (B.1.1.529) |
| Feb 22 | 0 | 0 | 0 | 0 | 0 | **59** | 0 | 0 | **59** | Omicron BA.1 |
| Mar 22 | 0 | 0 | 0 | 0 | 0 | 9 | 0 | 0 | 9 | Omicron BA.2 |
| Apr 22 | 0 | 0 | 0 | 0 | 0 | 1 | 0 | 0 | 1 | Omicron BA.2 |
| May 22 | 0 | 0 | 0 | 0 | 0 | 4 | 0 | 0 | 4 | Omicron BA.2 |
| Jun 22 | 0 | 0 | 0 | 0 | 0 | 22 | 0 | 0 | 22 | Omicron BA.2 |
| Jul 22 | 0 | 0 | 0 | 0 | 0 | 3 | 0 | 0 | 3 | Omicron (BA.4 and BA.5) |

*as per <https://users.math.msu.edu/users/weig/SARS-CoV-2_Mutation_Tracker.html>, <https://www.who.int/activities/tracking-SARS-CoV-2-variants>, and <https://cov-lineages.org/lineage_list.html>
