# Supplementary material for: SARS-CoV-2 variant survey: Comparison of RT-PCR screening with TGS and variant distribution across two divisions of Bangladesh
Source: PLoS One. 2024 Oct 17;19(10):e0311993. doi: 10.1371/journal.pone.0311993 (PMC11486398; doi:10.1371/journal.pone.0311993)
Supplement: S2 Fig — No significant difference was noted in terms of presence or absence of symptoms. (DOCX) [file pone.0311993.s004.docx]

**S3 Fig:**


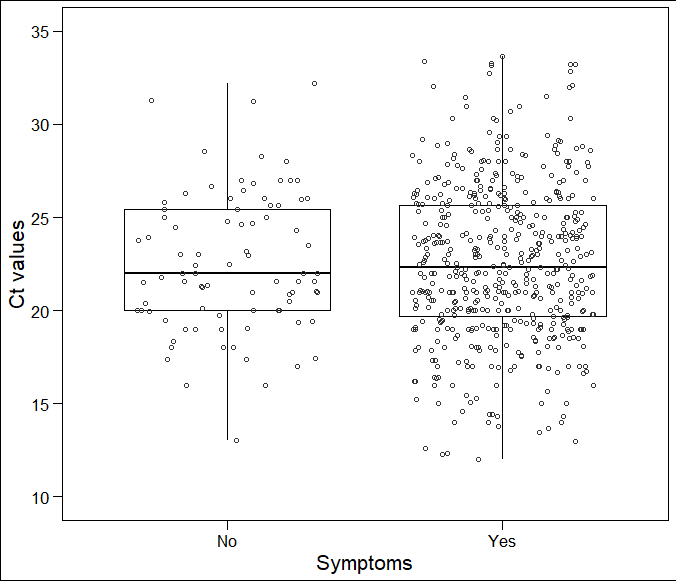


**Figure:** **Distribution of the Ct values of the NPS specimens in cases with and without symptoms of COVID-19.** No significant difference was noted in terms of presence or absence of symptoms.
