# Supplementary material for: SARS-CoV-2 variant survey: Comparison of RT-PCR screening with TGS and variant distribution across two divisions of Bangladesh
Source: PLoS One. 2024 Oct 17;19(10):e0311993. doi: 10.1371/journal.pone.0311993 (PMC11486398; doi:10.1371/journal.pone.0311993)
Supplement: S3 Fig — (A) Distribution of routine RT-PCR Ct values across variants. The significant differences in between the inter-variant median Ct-values were plotted. (B) Ct values of five primer-probes for each of the 600 samples. The Ct values of the mutant alleles ranged from 13 to 38, similar to routine RT-PCR Ct values. The variants were named according to the signals obtained from the variant PCR. The control mutation was D614G (not shown in the graph). (DOCX) [file pone.0311993.s005.docx]

**S4 (A) Fig:**


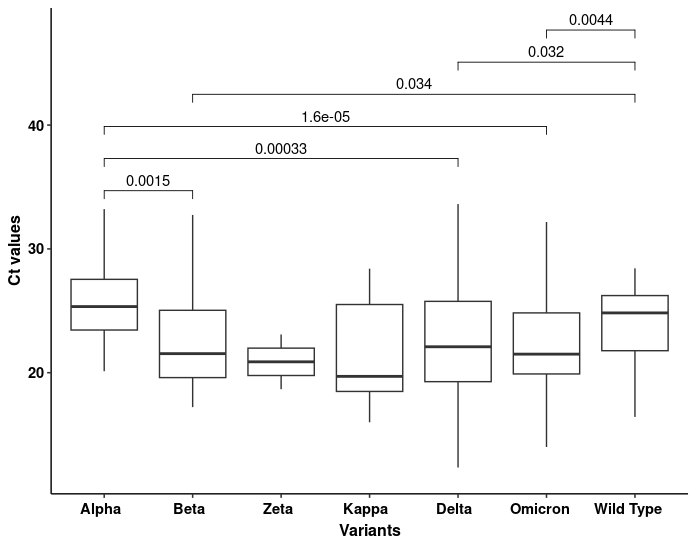


**Figure:** **Distribution of routine RT-PCR Ct values across variants.** The significant differences in between the inter-variant median Ct-values were plotted.

**S4 (B) Fig:**


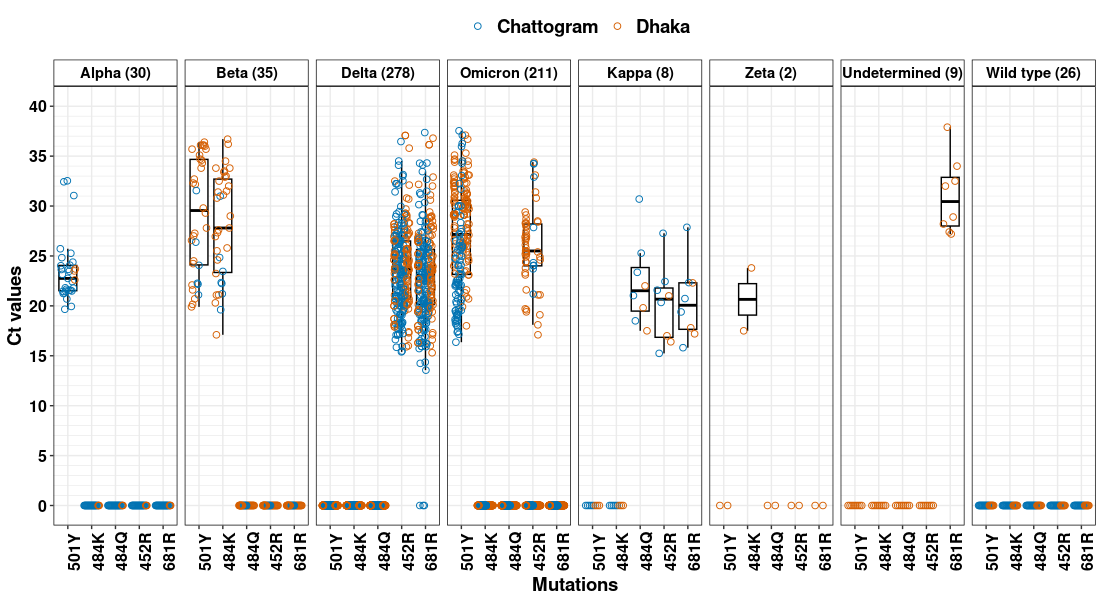


**Figure:** **Ct values of five primer-probes for each of the 600 samples.** The Ct values of the mutant alleles ranged from 13 to 38, similar to routine RT-PCR Ct values. The variants were named according to the signals obtained from the variant PCR. The control mutation was D614G (not shown in the graph).
